# Supplementary material for: Diagnostic Value of Middle Meatal Cultures versus Maxillary Sinus Culture in Acute and Chronic Sinusitis: A Systematic Review and Meta-Analysis
Source: J Clin Med. 2022 Oct 14;11(20):6069. doi: 10.3390/jcm11206069 (PMC9605498; doi:10.3390/jcm11206069)
Supplement: Supplementary file 1 [file jcm-11-06069-s001.zip › jcm-1853495-supplementary.pdf]

Supplementary Materials

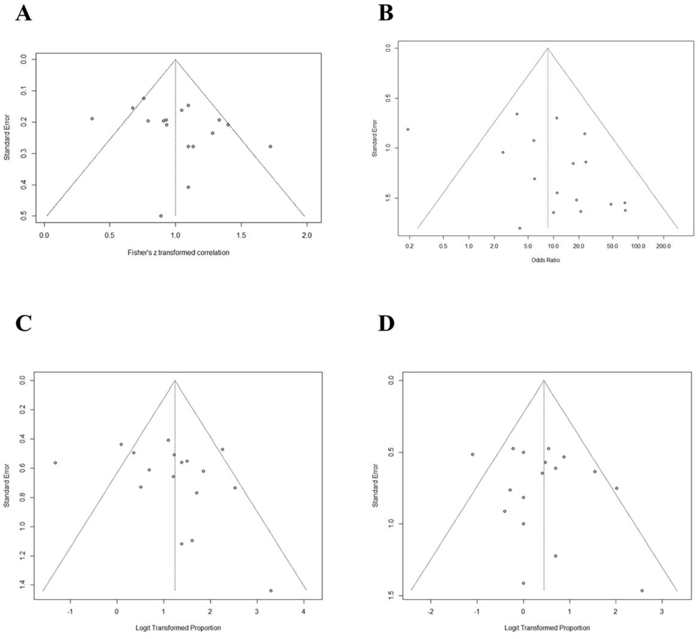

Figure S1. Begg funnel plots. Correlations between (**A**) middle meatus and maxillary sinus culture results. (**B**) Diagnostic odds ratio of middle meatus culture. (**C**) Sensitivity values. (**D**) Specificity values.

Table S1. Characteristics of the included studies.

Study	Number of Patients	Age, Median (Range) or Mean(SD), y	Age Group	Sex (Male/Female)	Nationality	Rhinosinusitis	Patient Conditions	Harvest Method	Isolate Number	Reference Test	r	TP	FN	FP	TN
Gold 1997	18	47 (25–72)	Adult	9/9	USA	Chronic	OP	suction tip	21	MS aspiration	0.857	13	0	2	6
Klossek 1998	533	42	Adult	300/233	France	Chronic	OP	swab	68	MS aspiration	0.64	48	5	7	8
Ferguson 1999	12	NA	Adult	NA	USA	Acute	OPD	suction tip	9	MS aspiration	0.8	5	1	1	2
Vogan 2000	13	44 (25–81)	Adult	NA	USA	Acute	OPD	swab	16	MS aspiration	0.938	11	2	1	2
Talbot 2001	46	37.6 (24–75)	Adult	27/26	USA	Acute	OPD	swab	45	MS aspiration	0.587	17	5	11	12
Casiano 2001	20	40 (23–77)	Adult	17/3	USA	Acute	Febrile ICU	tissue culture (biopsy)	29	MS aspiration	0.72	19	3	5	2
Kountakis 2002	18	50 (16–72)	Adult	12/6	Greece	Acute	Febrile ICU	swab	31	MS lavage	0.35	4	15	7	5
Joniau 2005	24	NA	Adult	NA	Belgium	Acute	OPD	glass micropipette	26	MS aspiration	0.885	16	4	0	6
Kirtsreesakul 2005	16	44±17	Adult	8/7	Thailand	Chronic	OP	aspiration instrument	16	MS aspiration	0.8125	8	4	1	3
Benninger 2006 (SAHP)	7	41.3	Adult	5/2	USA	Acute	OPD	swab	7	MS aspiration	0.71	4	1	1	1
Benninger 2006 (unpublished data)	26	NA	Adult	NA	USA	Acute	OPD	suction tip	26	MS aspiration	0.731	5	3	4	14
Araujo 2007	16	NA	Adult	NA	Brazil	Chronic	OPD	suction tip	16	MS aspiration	0.8	10	3	0	3
Hsin 2008	21	6.4 (2–12)	Children	12/9	Taiwan	Chronic	OPD	swab	41	MS aspiration	0.78	24	8	1	8
Hsin 2010	15	5.9 (3–11)	Children	9/6	Taiwan	Chronic	OPD	swab	29	MS aspiration	0.66	11	10	0	8
Hsin 2010	16	7.5 (4–10)	Children	10/6	Taiwan	Chronic	OPD	suction tip	30	MS aspiration	0.87	18	4	0	8
Thunberg 2013	30	45 (21–81)	Adult	6/24	Sweden	Acute	OPD	swab	30	MS aspiration	0.73	10	7	1	12
Szaleniec 2021	50	49 (19–83)	Adult	25/25	Poland	Chronic	OP	swab	50	MS swab	0.8	25	2	8	15

Abbreviations: NA, Not available; MS, maxillary sinus; OP, Operation; OPD, Outpatient department; ICU, Intensive care unit; TP, True positive; FP, False positive; FN, False Negative; TN, True negative.

Table S2. Methodological quality of the studies.

Reference	Risk of Bias				Concerns about Application		
	Patient Selection	Index Test	Reference Standard	Flow and Timing	Patient Selection	Index Test	Reference Standard
Gold 1997	Low	Low	Unclear	Unclear	Low	Low	Low
Klossek 1998	Low	Low	Low	Low	Low	Low	Low
Ferguson 1999	Low	Low	Unclear	Low	Low	Low	Low
Vogan 2000	Low	Low	Unclear	Low	Low	Low	Low
Talbot 2001	Low	Low	Unclear	Low	Low	Low	Low
Casiano 2001	Low	Low	Low	Low	Low	Low	Low
Kountakis 2002	Low	Low	Low	Low	Low	Low	Low
Joniau 2005	Unclear	Low	Unclear	Unclear	Low	Low	Low
Kirtsreesakul 2005	Low	Low	Low	Low	Low	Low	Low
Benninger 2006	Low	Low	Unclear	Unclear	Low	Low	Low
Araujo 2007	Unclear	Low	Unclear	Unclear	Low	Low	Low
Hsin 2008	Low	Low	Low	Low	Low	Low	Low
Hsin 2010	Low	Low	Unclear	Low	Low	Low	Low
Thunberg 2013	Low	Low	Unclear	Low	Low	Low	Low
Szaleniec 2021	Low	Low	Low	Low	Low	Low	Low
